# The Neurological Sequelae of Vitamin B12 Deficiency: A Systematic Review and Randomized Controlled Trial

**DOI:** 10.7759/cureus.83668

**Published:** 2025-05-07

**Authors:** Abir Abdalaal Hamza Ali, Fatima H.A. Mohamed, Samir Hago, Israa Fathelrahman Elsadig Elgali, Hager Elsir Sherfeldin Mohammed, Rania G Mirghani

**Affiliations:** 1 Internal Medicine, Maidstone Hospital, Maidstone, GBR; 2 Medicine, University Hospitals of North Midlands NHS Trust, Stoke on Trent, GBR; 3 Internal Medicine, Noble's Hospital, Douglas, IMN; 4 Neurology, Sheikh Jaber Al-Ahmad Al-Sabah Hospital, Kuwait City, KWT; 5 Internal Medicine, King Abdulaziz Medical City, Riyadh, SAU; 6 Internal Medicine/Neurology, Omdurman Teaching Hospital, Omdurman, SDN

**Keywords:** cobalamin, neurological sequelae, randomized controlled trials, systematic review, vitamin b12

## Abstract

Vitamin B12 deficiency is a well-recognized cause of neurological complications, including peripheral neuropathy, cognitive decline, and myelopathy. However, the extent of neurological benefits from supplementation, especially in subclinical cases, remains uncertain. This systematic review evaluated randomized controlled trials (RCTs) investigating the neurological effects of vitamin B12 supplementation. The review focused on treatment outcomes, route of administration, and population characteristics, along with the methodological quality of included studies.

Ten RCTs were included, involving individuals with clinical and subclinical vitamin B12 deficiency. Supplementation improved neurological symptoms in patients with overt deficiency, with oral therapy showing similar efficacy to intramuscular injections, better tolerability, and lower cost. In older adults with subclinical deficiency, supplementation did not significantly improve cognitive or neurological outcomes. In diabetic patients with neuropathy, improvements were noted in symptom scores, but not in objective neurological measures. Although reductions in homocysteine levels were observed, these biochemical changes did not consistently correlate with clinical improvements. Vitamin B12 supplementation is effective for patients with clinical deficiency but shows limited neurological benefit in subclinical cases. Further trials using standardized outcomes and longer follow-up are needed to refine treatment strategies and support biomarker-guided approaches.

## Introduction and background

Vitamin B12 (cobalamin) is an indispensable water-soluble micronutrient that serves as a cofactor for two critical enzymatic processes in humans as follows: methionine synthase, which facilitates the conversion of homocysteine to methionine - a reaction integral to DNA synthesis and epigenetic regulation - and methylmalonyl-CoA mutase, which catalyzes the isomerization of methylmalonyl-CoA to succinyl-CoA, a key step in mitochondrial energy metabolism [1]. These biochemical roles underpin its necessity for neuronal integrity, myelination, and neurotransmitter synthesis, rendering vitamin B12 deficiency a potent disruptor of neurological homeostasis [2]. Clinically, deficiency manifests as a spectrum of neurological sequelae, including peripheral neuropathy, cognitive impairment, myelopathy, and, in severe cases, irreversible neurodegeneration [3]. Despite its well-documented association with hematological abnormalities, the neurological consequences of B12 deficiency often present insidiously, complicating timely diagnosis and intervention, particularly in subclinical or early-stage cases.

The global burden of vitamin B12 deficiency is substantial, disproportionately affecting aging populations, individuals with gastrointestinal malabsorption disorders (e.g., pernicious anemia, Crohn’s disease), those adhering to plant-based diets devoid of animal-derived sources, and individuals using medications such as metformin and proton pump inhibitors (PPIs), which are emerging contributors to deficiency [4]. Emerging evidence suggests that even marginal deficiencies, previously deemed subclinical, may contribute to subtle neurocognitive dysfunction, raising concerns about long-term public health implications in an era of increasing dietary restrictions and aging demographics [5]. However, the pathophysiological mechanisms linking B12 insufficiency to neuronal injury remain incompletely elucidated, with hypotheses ranging from dysregulated myelination due to aberrant fatty acid metabolism to oxidative stress secondary to elevated homocysteine levels [6].

While observational studies and case reports have extensively characterized the neurological manifestations of B12 deficiency [7,8], high-quality evidence from randomized controlled trials (RCTs) - the gold standard for establishing causal relationships and therapeutic efficacy - remains fragmented. Existing systematic reviews often amalgamate heterogeneous study designs, obscuring the specific contributions of RCTs to understanding dose-response relationships, treatment modalities (e.g., oral vs. intramuscular supplementation), and the reversibility of neurological damage [9]. Furthermore, inconsistencies in diagnostic criteria for deficiency (e.g., serum B12 thresholds, methylmalonic acid/homocysteine biomarkers) and variability in neurological outcome measures across trials complicate meta-analytic synthesis and clinical translation.

This systematic review addresses these gaps by rigorously evaluating RCTs that investigate the neurological outcomes of vitamin B12 deficiency and its remediation. Specifically, we aimed to (1) synthesize evidence on the efficacy of B12 supplementation in reversing or mitigating neurological deficits, (2) delineate optimal therapeutic strategies (dosage, duration, route), and (3) assess the methodological quality of existing RCTs to identify biases and limitations. By focusing exclusively on RCTs, this review prioritizes causal inference and clinical applicability, offering evidence-based insights to guide neurology, nutrition, and primary care practice. In doing so, it also highlights critical avenues for future research, including the need for standardized diagnostic protocols and long-term outcome studies to resolve enduring controversies in B12-related neurology.

In an era where personalized medicine and preventive neurology are gaining traction, clarifying the role of B12 in neurological health is not merely academic - it is a pressing imperative for mitigating disability and improving quality of life in at-risk populations. This review seeks to bridge the chasm between biochemical theory and clinical practice, ensuring that therapeutic decisions are anchored in robust empirical evidence rather than historical precedent alone.

## Review

Methodology

Study Design and Aim

This systematic review was conducted in adherence to the Preferred Reporting Items for Systematic Reviews and Meta-Analyses (PRISMA) guidelines to ensure methodological transparency, rigor, and reproducibility [10]. The primary aim of this study was to evaluate the neurological sequelae of vitamin B12 deficiency and the efficacy of therapeutic interventions, as reported in RCTs.

Search Strategy and Information Sources

A systematic and exhaustive search strategy was developed to identify relevant studies across the electronic databases PubMed/MEDLINE, Scopus, Web of Science, and CINAHL. All these databases were searched for relevant literature from February 20 to February 27, 2025. To mitigate geographic and publication bias, grey literature sources - including clinical trial registries (ClinicalTrials.gov, WHO ICTRP), conference abstracts, and dissertations - were also screened. The search strategy combined Medical Subject Headings (MeSH) terms and free-text keywords related to vitamin B12 (e.g., “cobalamin,” “B12 deficiency”) and neurological outcomes (e.g., “neuropathy,” “myelopathy,” “cognitive impairment”). Boolean operators (AND, OR) and database-specific filters for RCTs were applied to refine results. A detailed Search Strategy for each database is provided in the table in the appendix. No restrictions were imposed on language or publication date; non-English studies were translated professionally to avoid exclusion bias.

Eligibility Criteria

Studies were selected using the Population, Intervention, Comparator, Outcomes, Study Design (PICOS) framework. The population of interest included individuals of any age with confirmed vitamin B12 deficiency, diagnosed via serum B12 levels (<200 pg/mL) or elevated biomarkers (methylmalonic acid >0.4 µmol/L, homocysteine >15 µmol/L). Interventions comprised vitamin B12 supplementation (any formulation, dose, or route of administration), while comparators included placebo, standard care, or alternative therapies. Primary outcomes focused on neurological improvements, such as changes in neurophysiological parameters (e.g., nerve conduction velocity), cognitive assessments (e.g., Mini-Mental State Examination {MMSE}), or clinical symptom resolution (e.g., paresthesia, gait abnormalities). Secondary outcomes included adverse events and dose-response relationships. Only peer-reviewed RCTs with a minimum follow-up duration of four weeks were eligible. Observational studies, case reports, non-randomized trials, and animal studies were excluded.

Study Selection and Data Extraction

Two independent reviewers screened titles, abstracts, and full-text articles with discrepancies resolved through discussion or arbitration by a third reviewer. Data extraction was performed using a standardized template, piloted on a subset of studies to ensure consistency. Extracted variables included study design, sample size, participant demographics, diagnostic criteria for B12 deficiency, intervention details, comparator, neurological outcome measures, adverse events, and funding sources. Corresponding authors were contacted to clarify ambiguities or request unreported data.

Risk of Bias Assessment

The risk of bias of all included RCTs was independently assessed using the Cochrane Risk of Bias 2 (RoB 2) tool [11]. This tool evaluates the following five key domains: bias arising from the randomization process, bias due to deviations from intended interventions, bias due to missing outcome data, bias in measurement of the outcome, and bias in selection of the reported result. Each domain was rated as "low risk," "some concerns," or "high risk," and an overall judgment was derived for each study. The assessment was guided by the available methodological information in each study, including blinding procedures, completeness of outcome data, and the transparency of reporting. Disagreements were resolved through consultation with a third reviewer who served as a tiebreaker.

Data Synthesis and Analysis

A narrative synthesis approach was employed to analyze and interpret findings, structured by neurological domains (e.g., peripheral neuropathy, cognitive dysfunction) and intervention characteristics (e.g., route of administration, treatment duration). Findings were synthesized thematically to address the review’s objectives, with results presented in summary tables and figures to enhance clarity.

Ethical Considerations

As this review synthesized data from publicly available studies, formal ethical approval was not required. However, ethical integrity was maintained by accurately representing original findings, transparently disclosing conflicts of interest, and avoiding selective reporting of outcomes.

Results

Study Selection Process

**Figure 1:**
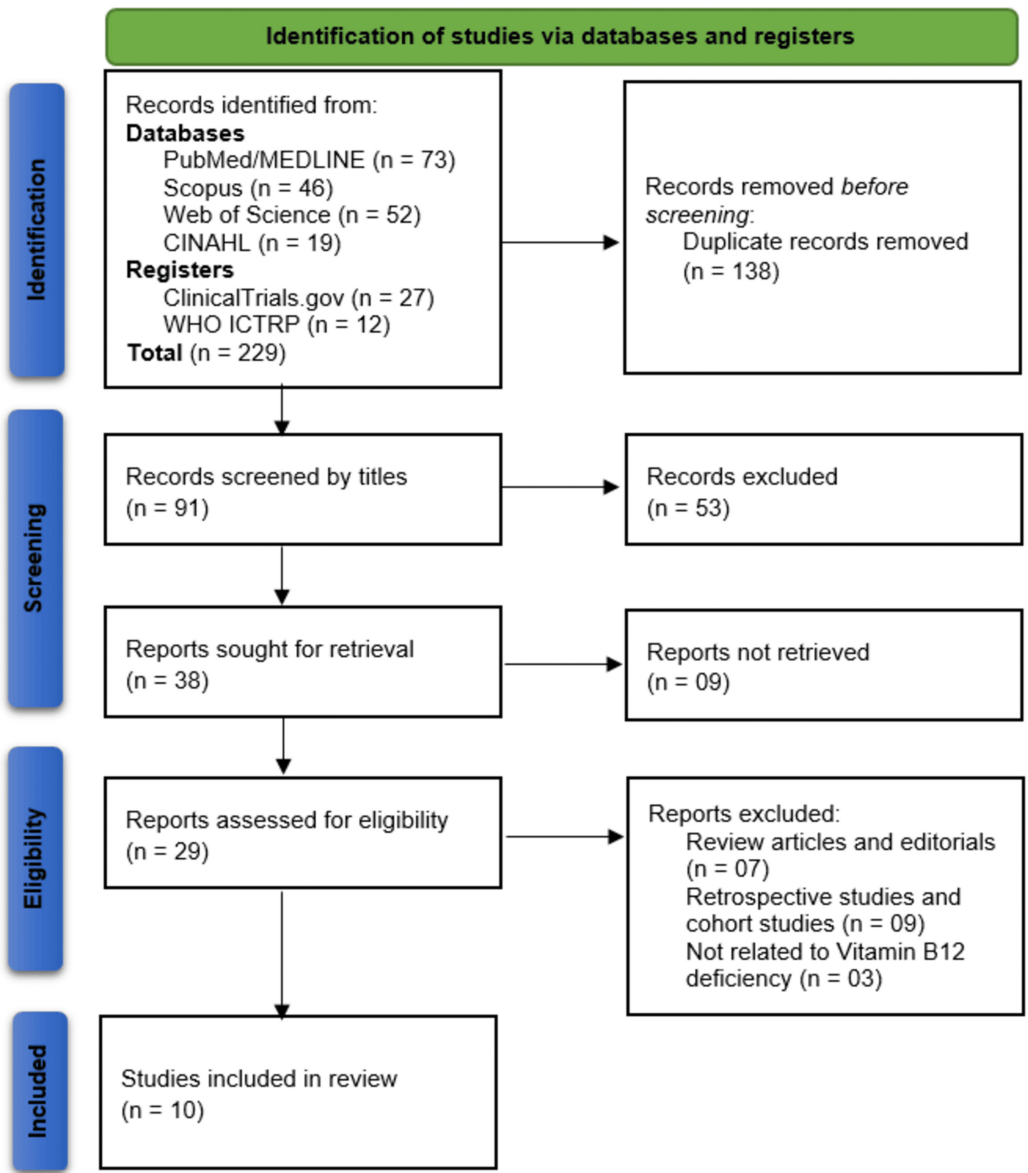
Representation of studies selection through PRISMA flowchart. PRISMA: Preferred Reporting Items for Systematic Reviews and Meta-Analyses

The systematic search identified a total of 229 records from multiple sources as follows: PubMed/MEDLINE (n=73), Scopus (n=46), Web of Science (n=52), CINAHL (n=19), ClinicalTrials.gov (n=27), and WHO ICTRP (n=12). After removing 138 duplicate records, 91 studies underwent title screening, resulting in the exclusion of 53 irrelevant records. Of the remaining 38 reports sought for retrieval, nine were unavailable, leaving 29 studies for full-text eligibility assessment. Among these, 19 were excluded (seven review articles/editorials, nine retrospective/cohort studies, and three unrelated to vitamin B12 deficiency), culminating in 10 studies being included in the final review. The selection process is detailed in the PRISMA flowchart (Figure 1).

Summary of Studies Included

A total of 10 RCTs were included in this systematic review, with sample sizes ranging from 9 to 2919 participants [12-21]. These studies were conducted across various populations, predominantly older adults aged 65 years and above, individuals with vitamin B12 deficiency, and patients with type 2 diabetes experiencing peripheral neuropathy. Intervention strategies varied across studies. Most trials utilized oral vitamin B12 supplementation in doses ranging from 10 µg to 2 mg per day, either alone or in combination with other micronutrients such as folic acid and vitamin B6. Two trials compared oral versus intramuscular routes of cobalamin administration and found both to be equally effective in improving hematological and neurological parameters, with oral formulations demonstrating better tolerability and cost-effectiveness [12,20].

Several trials examined neurological or cognitive outcomes in elderly participants with subclinical B12 deficiency. Dangour et al., de Koning et al., and Eussen et al. reported limited or no significant neurological or cognitive benefits following daily oral B12 supplementation, even over extended durations [13-16]. However, a slight improvement in health-related quality of life was noted in one study [15]. In contrast, studies involving patients with existing neuropathic symptoms yielded more favorable outcomes. Franques et al. observed rapid neurological improvements in a small cohort with B12-responsive neuropathy [19]. Farvid et al. and Fonseca et al. demonstrated significant improvements in subjective neuropathy scores following supplementation with B12-containing micronutrient combinations, although objective electrophysiological measures remained largely unchanged [17,18]. One large-scale trial found no long-term benefit of methylcobalamin and folic acid supplementation on cognitive decline in older adults with mild cognitive impairment, although transient improvements at 12 months were observed [21].

The included studies showed heterogeneity in participant selection, interventions, outcome measures, and study durations, which limits direct comparisons. However, the findings collectively highlight that while vitamin B12 supplementation may be beneficial in symptomatic individuals or those with overt deficiency, its preventive or cognitive-enhancing effects in asymptomatic, mildly deficient elderly populations remain inconclusive (Table 1).

**Table 1:** Characteristics of the included studies. IM: intramuscular; PO: per os (oral administration); Hb: hemoglobin; MCV: mean corpuscular volume; WBC: white blood cell; B12: vitamin B12 (cobalamin); CMAP: compound muscle action potential; GDS-15: Geriatric Depression Scale-15 items; HR-QoL: Health-Related Quality of Life; SF-12: Short Form-12 Health Survey; EQ-5D: EuroQol 5-dimensions; VAS: visual analog scale; MV: multivitamin; MVB: multivitamin with B complex; MNSI: Michigan Neuropathy Screening Instrument; LMF-MC-PLP: L-methylfolate, methylcobalamin, pyridoxal-5′-phosphate; VPT: Vibration Perception Threshold; NTSS-6: Neuropathy Total Symptom Score-6; MMA: methylmalonic acid; ONLS: Overall Neuropathy Limitations Scale; CDR_SOB: Clinical Dementia Rating-Sum of Boxes; MCI: mild cognitive impairment

Author	Publishing year	Study design	Sample size	Study population	Intervention	Comparator	Outcome	Key findings
Bolaman et al. [12]	2003	Randomized control trial	60 patients	Patients aged ≥16 years with megaloblastic anemia due to cobalamin deficiency	Oral cobalamin 1000 μg daily for 10 days, then weekly for 4 weeks, then monthly for life	Intramuscular cobalamin 1000 μg daily for 10 days, then weekly for 4 weeks, then monthly for life	Hematologic parameters (Hb, MCV, WBC, Platelets), serum B12 levels, neurologic symptoms, tolerability, and cost	PO cobalamin was as effective as IM for hematologic and neurologic improvement, with better tolerance and lower cost; both showed reticulocytosis and neurologic gains; longer-term studies are needed due to small sample size
Dangour et al. [13]	2011	Randomized double-blind placebo-controlled trial	200	Older adults aged ≥75 years with biochemical vitamin B12 insufficiency but without anaemia	Daily oral tablet containing 1 mg vitamin B12	Matching placebo tablet	Change in electrophysiological indices of neurosensory function after 12 months	Given the high rate of vitamin B12 deficiency in later life, this trial may hold important public health value
Dangour et al. [14]	2015	Double-blind, randomized, placebo-controlled trial	201 (191 completed)	Older adults aged ≥75 years with moderate vitamin B12 deficiency (107-210 pmol/L), without anemia or neurologic/cognitive symptoms	1 mg daily oral crystalline vitamin B12 for 12 months	Placebo	Neurologic function (e.g., posterior tibial CMAP amplitude), central motor conduction, cognitive function, clinical neurological exam	Vitamin B12 supplementation shows no significant neurologic or cognitive benefit in moderately deficient older adults without anemia or symptoms
De Koning et al. [15]	2016	Randomized controlled trial	2919	Older adults (≥65 years) with homocysteine ≥12 µmol/L	Daily supplementation of 500 µg vitamin B12 + 400 µg folic acid + 15 µg vitamin D3 for 2 years	Placebo (with 15 µg vitamin D3)	Depressive symptoms (GDS-15), HR-QoL (SF-12 Mental/Physical, EQ-5D Index and VAS)	Lowering homocysteine did not reduce depressive symptoms but had a small positive effect on EQ-5D index score (p=0.004)
Eussen et al. [16]	2006	Double-blind, placebo-controlled randomized trial	195	Individuals aged ≥70 years with mild vitamin B12 deficiency	1) 1000 μg vitamin B12 daily; 2) 1000 μg vitamin B-12 + 400 μg folic acid daily for 24 weeks	Placebo	Cognitive function assessed using a neuropsychologic test battery (attention, construction, sensomotor speed, memory, executive function)	No significant improvement in cognitive function with supplementation. Memory function improved more in the placebo group than the vitamin B12 alone group (p=0.0036)
Farvid et al. [17]	2011	Randomized, double-blind, placebo-controlled clinical trial	75 (67 completed)	Type 2 diabetes patients	Group MV: zinc (20 mg), magnesium (250 mg), vitamin C (200 mg), vitamin E (100 IU); Group MVB: MV + vitamin B1 (10 mg), B2 (10 mg), B6 (10 mg), biotin (200 μg), B12 (10 μg), folic acid (1 mg)	Placebo	Neuropathy indices (MNSI questionnaire and exam), glycemic control, capillary blood flow, electrophysiological measures	MNSI questionnaire scores significantly improved in both MV and MVB groups, most notably in MVB. No significant improvements in objective neuropathy exams, glycemic control, or electrophysiological measures compared to placebo
Fonseca et al. [18]	2013	Multicenter, randomized, double-blind, placebo-controlled trial	214	Patients with type 2 diabetes and sensory neuropathy (VPT: 25-45 volts)	L-methylfolate 3 mg, methylcobalamin 2 mg, pyridoxal-5′-phosphate 35 mg (LMF-MC-PLP) for 24 weeks	Placebo	Primary: Vibration Perception Threshold (VPT); Secondary: NTSS-6, SF-36, plasma levels of folate, B6, B12, MMA, homocysteine	No significant change in VPT. Significant improvement in NTSS-6 at weeks 16 and 24. Homocysteine levels decreased. Quality of life improved. Few adverse events noted
Franques et al. [19]	2019	Randomized control trial	9	Patients with B12-responsive neuropathy	Vitamin B12 supplementation	None	Improvement in Overall Neuropathy Limitations Scale (ONLS) score	Four patients had low serum B12 levels. Six patients showed improvement in less than 1 month. B12-responsive neuropathy is heterogeneous, and B12 deficiency should be ruled out in idiopathic or sensory neuronopathy cases
Kuzminski et al. [20]	1998	Randomized controlled trial	38 newly diagnosed cobalamin-deficient patients	Cobalamin-deficient patients	2 mg oral cyanocobalamin daily for 120 days	1 mg intramuscular cyanocobalamin on specified days	Hematologic and neurologic improvement, serum cobalamin, methylmalonic acid, and homocysteine levels	Oral cyanocobalamin (2 mg daily) was as effective as parenteral cyanocobalamin (1 mg intramuscularly) with significantly higher serum cobalamin and lower methylmalonic acid levels at 4 months post-treatment
Kwok et al. [21]	2020	Randomized control trial	279	Older MCI patients (≥65 years) with serum homocysteine ≥10.0 μmol/L	Methylcobalamin 500 μg + folic acid 400 μg daily	Placebo tablets	Cognitive decline (CDR_SOB), executive function, depressive symptoms	No cognitive decline difference at 24 months. The supplement group improved at 12 months but not at 24 months. Aspirin use negatively interacted with B vitamins on cognitive function

Risk of Bias Assessment Results

**Figure 2:**
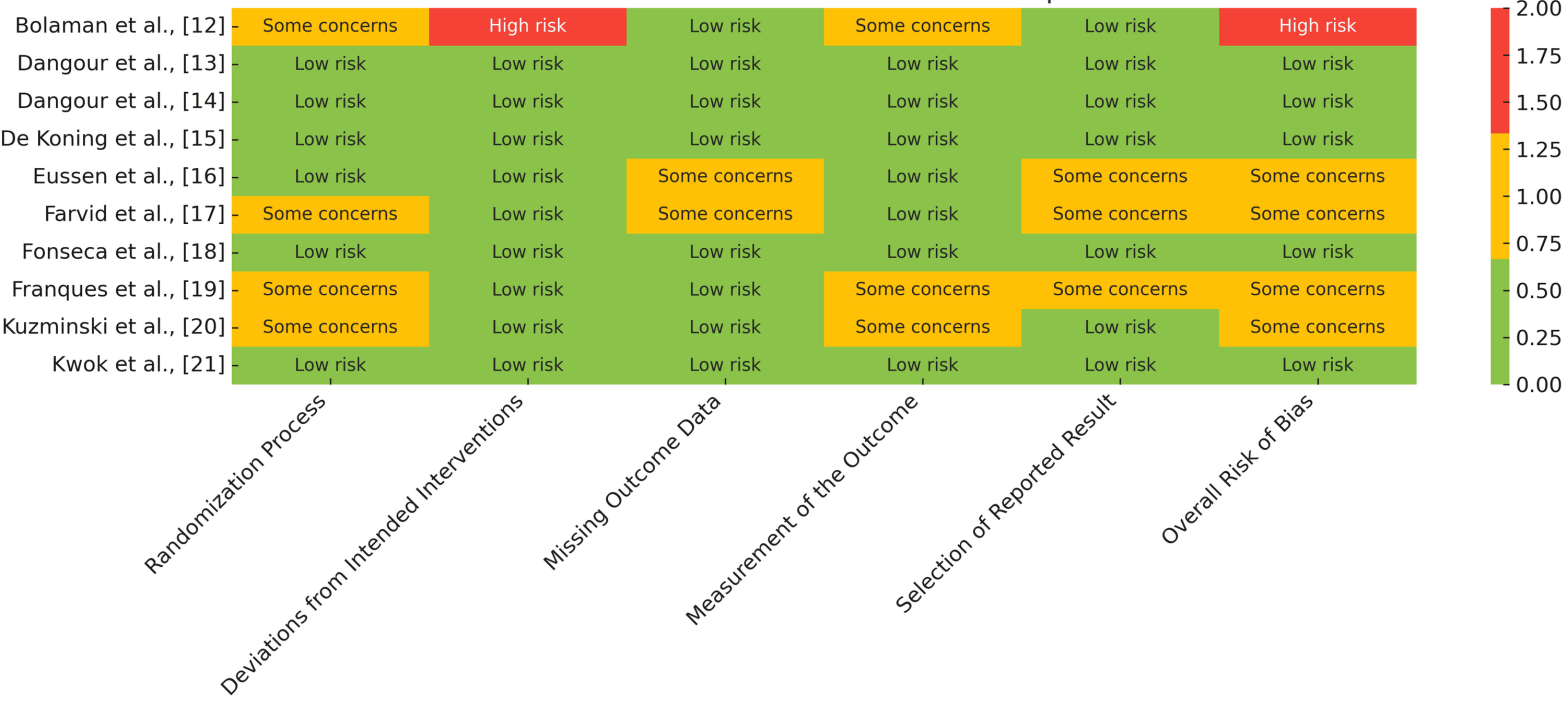
Risk of bias assessment using Cochrane ROB 2 tool. Green (low risk): indicates that the study met criteria for low risk of bias in the respective domain; amber (some concerns): indicates potential issues or unclear methods that may introduce bias; red (high risk): indicates a high likelihood of bias that could affect the study's outcomes ROB2: risk of bias 2

Of the 10 included RCTs, five were judged to have a low overall risk of bias [13-15,18,21]. Four studies had some concerns, mainly due to unclear randomization methods, incomplete reporting, or potential bias in outcome measurement [16-19,20]. One study was rated as having a high risk of bias due to its open-label design and potential performance bias [12]. These findings indicate variability in methodological rigor among the included trials, which may influence the reliability of their reported outcomes (Figure 2).

Discussion

Vitamin B12 deficiency has long been recognized for its hematologic implications, but its neurological consequences are increasingly receiving attention due to their potential to cause irreversible damage if left untreated. This systematic review aimed to synthesize evidence from RCTs evaluating the effects of vitamin B12 supplementation on neurological outcomes. The review included 10 high-quality RCTs encompassing a wide variety of populations, intervention regimens, and outcome measures. Despite biological plausibility and observational associations between low B12 and neurological impairment, the evidence from RCTs remains mixed and, at times, inconclusive.

Several RCTs provided robust support for the neurological benefits of vitamin B12 in patients with clinical deficiency, particularly in those with megaloblastic anemia or overt neuropathy. Bolaman et al. and Kuzminski et al. both reported equivalent efficacy between oral and intramuscular vitamin B12 administration in correcting neurological symptoms, with the oral route showing better tolerability and cost-effectiveness [12,20]. On the other hand, trials involving older adults with subclinical or biochemical B12 insufficiency but without anemia or symptoms failed to demonstrate significant improvements in cognitive or neurological outcomes. This contrast highlights the heterogeneity in study populations and the potential role of baseline deficiency severity in determining treatment efficacy.

Efficacy of Oral Versus Intramuscular B12 Supplementation

Historically, parenteral administration was considered the gold standard for treating vitamin B12 deficiency due to concerns about malabsorption with oral formulations. However, two pivotal trials included in this review challenge this long-standing view. Bolaman et al. randomized 60 patients with megaloblastic anemia to receive either oral or intramuscular vitamin B12 and found no significant difference in hematological or neurological outcomes between the two routes [12]. Similarly, Kuzminski et al., in a study of 38 cobalamin-deficient patients, demonstrated that oral cyanocobalamin (2 mg/day) was as effective as intramuscular injections, with better biochemical markers such as higher serum B12 and lower methylmalonic acid levels at follow-up [20].

These findings suggest that oral B12 may be a viable and more patient-friendly option, especially for long-term management. The implications are particularly significant in low-resource settings where access to injections and trained personnel may be limited [22]. However, both studies were limited by small sample sizes and lacked long-term follow-up, necessitating larger multicenter trials to confirm these findings across broader populations.

Subclinical Deficiency and Lack of Neurological Improvement

One of the most striking findings across several studies was the lack of neurological or cognitive improvement in older adults with subclinical or moderate B12 deficiency. Dangour et al. conducted two large RCTs involving adults aged ≥75 years with biochemical B12 deficiency but no anemia or symptoms [13,14]. Both trials reported no significant changes in neurological function, electrophysiological parameters, or cognitive performance after 12 months of 1 mg/day oral B12 supplementation. Similarly, Eussen et al. found no improvement in cognitive domains following B12 supplementation, and in some cases, placebo groups fared better, as seen with memory performance [16].

These results call into question the clinical benefit of screening and treating mild or asymptomatic B12 deficiency in the elderly. While observational studies have linked low serum B12 levels to cognitive decline, RCT evidence suggests that supplementation does not necessarily translate into functional improvements [23]. One possible explanation is the presence of irreversible neuronal damage by the time B12 deficiency is detected biochemically, especially in the absence of anemia or symptoms [24]. Another consideration is the multifactorial nature of cognitive impairment in the elderly, where vitamin B12 may play only a minor role.

Homocysteine as a Mediator and Surrogate Marker

Several studies explored the hypothesis that elevated homocysteine, a known neurotoxin and cardiovascular risk factor, mediates the neurological effects of B12 deficiency. De Koning et al. evaluated over 2900 older adults with homocysteine ≥12 µmol/L and found that a two-year regimen of B12, folic acid, and vitamin D3 significantly reduced homocysteine but did not lead to improvements in depressive symptoms or most quality of life measures [15]. Fonseca et al. also observed significant reductions in homocysteine and improvements in subjective neuropathy scores, but not in objective neurological tests [18].

This suggests that while homocysteine levels may be modifiable through supplementation, the clinical relevance of this biochemical change remains uncertain. Homocysteine may act more as a surrogate marker rather than a causal agent in neurological dysfunction, or its effects may be too subtle or delayed to be captured within the duration of most trials [25]. The lack of correlation between homocysteine reduction and clinical outcomes underscores the need for more targeted biomarkers and longer follow-up periods in future studies.

Vitamin B12 in Diabetic Neuropathy

Three studies evaluated the role of B12 in diabetic populations with neuropathy, a condition often exacerbated by metformin use, which is known to deplete B12 stores. Farvid et al. investigated micronutrient supplementation in type 2 diabetes and found significant improvements in MNSI questionnaire scores, particularly in the group receiving B12 in combination with other B vitamins [17]. However, objective measures of neuropathy, such as electrophysiological tests and capillary blood flow, did not significantly change. Fonseca et al. similarly reported improvements in NTSS-6 symptom scores and quality of life indices but not in vibration perception threshold or nerve conduction parameters [18].

These results highlight a key challenge in diabetic neuropathy research-subjective improvements in symptoms may not always align with measurable changes in nerve function. It is possible that patient-reported outcomes reflect reduced neuropathic pain or paresthesia that do not correspond to changes in nerve conduction. Moreover, the use of combination therapies in these trials complicates the attribution of benefit to vitamin B12 specifically. However, the consistent trend toward symptom relief in diabetic neuropathy suggests a potential supportive role for B12 supplementation in this population.

Rapid Response in B12-Responsive Neuropathy

The case series by Franques et al., though small in sample size (n=9), offered valuable insights into B12-responsive neuropathy [19]. The study reported rapid improvements in the Overall Neuropathy Limitations Scale (ONLS) in six patients within one month of intramuscular (IM) B12 therapy, underscoring the potential for rapid neurological recovery in properly identified cases. The heterogeneity observed in the response further emphasized the need for individualized evaluation and the importance of ruling out B12 deficiency in patients with idiopathic or sensory neuronopathy.

This evidence supports the clinical practice of empiric B12 therapy in suspected neuropathic syndromes, especially when diagnostic ambiguity exists [26]. Electrophysiological improvement and functional gains in such responsive individuals argue strongly in favor of prompt and adequate replacement therapy, even in the absence of clear biochemical evidence of deficiency.

Cognitive Decline and Vitamin B12

The largest trial focusing on cognitive outcomes, conducted by Kwok et al., included 279 older patients with mild cognitive impairment (MCI) and elevated homocysteine levels [21]. Although the supplement group showed transient improvements at 12 months, these gains were not sustained at 24 months. The study also reported a potential interaction between aspirin use and vitamin B efficacy, suggesting that concurrent medications may modulate treatment effects.

These findings align with other RCTs suggesting that vitamin B12 supplementation may not reverse or prevent cognitive decline, especially in individuals with established neurodegenerative changes or in cases where treatment is delayed. It also raises questions about the appropriate duration of supplementation trials and the need to account for confounding variables such as polypharmacy, which is common in the elderly.

Limitations

While the included RCTs provide valuable insights, several limitations must be acknowledged. First, heterogeneity in study design, populations, dosages, and outcome measures complicates cross-trial comparisons. Second, the duration of most interventions may have been insufficient to capture long-term neurological changes, particularly in neurodegenerative or chronic conditions. Third, many trials relied on subjective outcome measures or limited neurophysiological assessments, reducing the objectivity and generalizability of findings.

Furthermore, adherence to supplementation, baseline nutritional status, comorbidities, and genetic polymorphisms affecting B12 metabolism were not uniformly controlled or reported across studies. These confounders may have diluted treatment effects or obscured subgroup benefits. Additionally, the use of combination supplements in some trials introduces attribution bias, as the individual contributions of B12 cannot be isolated.

Clinical and Public Health Implications

Despite the mixed evidence, certain clinical messages emerge clearly. For patients with overt vitamin B12 deficiency - characterized by anemia, elevated methylmalonic acid or homocysteine, and neurological symptoms - prompt supplementation leads to both hematological and neurological recovery. The choice of oral versus intramuscular route can be guided by patient preference, tolerability, and access to care.

In asymptomatic individuals with biochemical B12 deficiency, particularly the elderly, the decision to supplement should be individualized, weighing potential benefits against costs and pill burden. Public health strategies focusing on screening may need to be refined to target those most at risk or likely to benefit. Routine supplementation in the absence of clinical symptoms or objective neurological decline is not supported by current evidence.

Future Directions

Future research should aim for larger, well-powered RCTs with longer follow-up durations and standardized outcome measures. Biomarker-guided stratification may help identify subgroups most likely to benefit from B12 supplementation. Trials investigating the interaction between B12 and other micronutrients, medications, and genetic factors will also be crucial in developing personalized treatment strategies.

Moreover, there is a need for neuroimaging and advanced electrophysiological studies to better understand the mechanistic pathways of B12-related neurological damage and recovery. Finally, implementation studies exploring the cost-effectiveness and acceptability of oral versus intramuscular regimens across different healthcare systems can inform guideline development and policy decisions.

## Conclusions

This review highlights that vitamin B12 supplementation offers clear neurological benefits in individuals with clinically evident deficiency, such as those with megaloblastic anemia or peripheral neuropathy, with both oral and intramuscular routes proving equally effective. However, in cases of subclinical deficiency -particularly among older adults without anemia or neurological symptoms - the evidence does not support significant cognitive or neurological improvement. While subjective symptom relief was noted in diabetic neuropathy, objective measures remained unchanged, and reductions in homocysteine levels did not consistently correlate with clinical outcomes. These findings underscore the importance of targeted supplementation in symptomatic individuals, while also highlighting the need for early identification and intervention in at-risk populations, rather than waiting for symptoms to manifest, especially given the long half-life of vitamin B12. Future research should focus on long-term outcomes, more sensitive neurological assessments, and stratification based on biochemical markers to optimize clinical decision-making and improve patient care.

## Appendices

**Table 2:** Search strategies for different databases.

Sr. no	Database	Search strategy
1	PubMed/MEDLINE	("Vitamin B12 Deficiency"[Mesh] OR "vitamin B12 deficiency" OR "cobalamin deficiency" OR "B12 deficiency") AND ("Nervous System Diseases"[Mesh] OR "neurological symptoms" OR "neurological manifestations" OR "neurological disorders" OR "neuropathy" OR "cognitive impairment" OR "dementia" OR "myelopathy") AND ("Randomized Controlled Trial"[Publication Type] OR "randomized controlled trial" OR "RCT" OR "clinical trial")
2	Scopus	TITLE-ABS-KEY("vitamin B12 deficiency" OR "cobalamin deficiency" OR "B12 deficiency") AND TITLE-ABS-KEY("neurological sequelae" OR "neurological manifestations" OR "neurological symptoms" OR "neuropathy" OR "cognitive impairment" OR "dementia" OR "myelopathy") AND TITLE-ABS-KEY("randomized controlled trial" OR "RCT" OR "clinical trial")
3	Web of Science	TS=("vitamin B12 deficiency" OR "cobalamin deficiency" OR "B12 deficiency") AND TS=("neurological sequelae" OR "neurological symptoms" OR "neurological manifestations" OR "neurological disorders" OR "neuropathy" OR "cognitive decline" OR "dementia" OR "myelopathy") AND TS=("randomized controlled trial" OR "RCT" OR "clinical trial")
4	CINAHL (via EBSCOhost)	("vitamin B12 deficiency" OR "cobalamin deficiency" OR "B12 deficiency") AND ("neurological sequelae" OR "neurological symptoms" OR "neurological manifestations" OR "neuropathy" OR "cognitive impairment" OR "dementia" OR "myelopathy") AND ("randomized controlled trial" OR "RCT" OR "clinical trial")
